# Identification of Candidate Genes for Dyslexia Susceptibility on Chromosome 18

**DOI:** 10.1371/journal.pone.0013712

**Published:** 2010-10-28

**Authors:** Thomas S. Scerri, Silvia Paracchini, Andrew Morris, I. Laurence MacPhie, Joel Talcott, John Stein, Shelley D. Smith, Bruce F. Pennington, Richard K. Olson, John C. DeFries, Anthony P. Monaco

**Affiliations:** 1 Wellcome Trust Centre for Human Genetics, University of Oxford, Oxford, United Kingdom; 2 School of Life and Health Sciences, Aston University, Birmingham, United Kingdom; 3 Department of Physiology, University of Oxford, Oxford, United Kingdom; 4 Department of Pediatrics and Munroe Meyer Institute, University of Nebraska Medical Center, Omaha, Nebraska, United States of America; 5 Department of Psychology, University of Denver, Denver, Colorado, United States of America; 6 Institute for Behavioral Genetics, University of Colorado, Boulder, Colorado, United States of America; Harvard University, United States of America

## Abstract

**Background:**

Six independent studies have identified linkage to chromosome 18 for developmental dyslexia or general reading ability. Until now, no candidate genes have been identified to explain this linkage. Here, we set out to identify the gene(s) conferring susceptibility by a two stage strategy of linkage and association analysis.

**Methodology/Principal Findings:**

Linkage analysis: 264 UK families and 155 US families each containing at least one child diagnosed with dyslexia were genotyped with a dense set of microsatellite markers on chromosome 18. Association analysis: Using a discovery sample of 187 UK families, nearly 3000 SNPs were genotyped across the chromosome 18 dyslexia susceptibility candidate region. Following association analysis, the top ranking SNPs were then genotyped in the remaining samples. The linkage analysis revealed a broad signal that spans approximately 40 Mb from 18p11.2 to 18q12.2. Following the association analysis and subsequent replication attempts, we observed consistent association with the same SNPs in three genes; melanocortin 5 receptor (*MC5R*), dymeclin (*DYM*) and neural precursor cell expressed, developmentally down-regulated 4-like (*NEDD4L*).

**Conclusions:**

Along with already published biological evidence, *MC5R*, *DYM* and *NEDD4L* make attractive candidates for dyslexia susceptibility genes. However, further replication and functional studies are still required.

## Introduction

Developmental dyslexia (DD [MIM 127700]) is a specific impairment in reading ability not directly related to intelligence, socio-economic background, general motivation, educational opportunity or sensory acuity [1], [2], [3], [4], [5], [6]. The prevalence of DD is reported as 5% to 17.5% [1], [2], [3], [4], [5]. Familial observations [7], [8], [9], [10] and twin studies [11], [12], [13] suggest a genetic aetiology of DD, and the heritability has been estimated between 0.30 and 0.70 [13], [14], [15], [16]. Recently, genetic studies have begun to unravel this complex aetiology.

Linkage studies of DD susceptibility have identified nine loci of interest, named *DYX1* to *DYX9* [2], [3], [5]. Candidate DD susceptibility genes have since been identified close to some of these loci. Firstly, “dyslexia susceptibility 1 candidate 1” (*DYX1C1* [MIM 608706]) was found close to *DYX1* on chromosome 15 [17]. Both *KIAA0319* (MIM 609269) and “doublecortin domain containing protein 2” (*DCDC2* [MIM 605755]) were indentified at *DYX2* on chromosome 6 [18], [19], [20]. The three genes “family with sequence similarity 176, member A” (*FAM176A*), “mitochondrial ribosomal protein L19” (*MRPL19* [MIM 611832]) and “chromosome 2 open reading frame 3” (*C2ORF3* [MIM 189901]) have been implicated at *DYX3* on chromosome 2 [21]. Finally, “roundabout, axon guidance receptor, homolog 1 (Drosophila)” (*ROBO1* [MIM 602430]) has been found close to *DYX5* (MIM 606896) on chromosome 3 [22].

We performed the first two quantitative-trait locus (QTL) based genome-wide linkage screens for DD using 89 United Kingdom (UK) and 119 United States (US) families [23]. Both revealed their most significant QTLs at chromosome 18p11.2 (*DYX6* [MIM 606616]), with various reading-related measures. An independent set of 84 UK families replicated this linkage at 18p11.2, and also revealed linkage at 18q12.2.

We then performed a multi-variate linkage study with the original 89 UK families to explore the contribution of six different reading-related traits to *DYX6* [24]. Dropping any one of these reading-related measures from the multi-variate model significantly reduced the linkage at *DYX6*, thereby implying that all measures were influenced by the underlying QTL.

Seven independent studies have failed to observe linkage at 18p11.2 with DD [25], [26], [27], [28], [29], [30], [31]. However, a study of 82 German families has observed a weak signal at 18p11 (LOD≈0.5) and 18q12 (LOD≈0.6) [32] with different reading-related traits, and a study of 108 Dutch families observed linkage to 18q12–q21 with DD (LOD = 2.0) [33]. Furthermore, a study of 403 Australian twin-families found strongest genome-wide linkage for reading ability at 18p11.2 (LOD = 1.70) and 18q12.1 (LOD = 2.0) [34]. Finally, the Framingham study of 705 stroke- and dementia-free individuals found strongest genome-wide linkage for reading ability at 18p11.2 (LOD = 5.0) [35].

Here, we conduct linkage analysis by genotyping our UK and US families to an approximate density of 1 microsatellite marker every 5 cM. A further 91 UK and 39 US families were also similarly genotyped. These new families continue to support linkage to chromosome 18. Subsequently, we performed a high-density association study by genotyping nearly 3000 SNPs from 18p11.31 to 18q21.31, covering the linkage region, in a discovery sample of 187 of the UK families. This yielded significant results in numerous genes, of which we could replicate several in our remaining samples of 102 UK families, 152 US families and 317 UK DD cases. In particular, we find consistent associations within the genes “melanocortin 5 receptor” (*MC5R* [MIM 600042]), dymeclin (*DYM* [MIM 607461]) and “neural precursor cell expressed, developmentally down-regulated 4-like” (*NEDD4L* [MIM 606384]).

## Materials and Methods

### Ethics Statement

Ethical approval for this study for the UK samples was acquired from the Oxfordshire Psychiatric Research Ethics Committee (OPREC O01.02). Written informed consent to participate in this study was obtained from all individuals prior to taking blood or buccal samples for DNA extraction, with the understanding that they could withdraw from the study at any time. Research plans and consent forms for the US families were reviewed and approved by the Institutional Review Boards of both the University of Colorado and the University of Nebraska Medical Center.

### Sample collection

The UK families were identified at clinics and schools of the Berkshire area, and have been detailed previously [23]. Families were ascertained if the proband had a British abilities scales (BAS) single-word reading score >2 standard deviations (SDs) below that predicted by their intelligence quotient (IQ) derived from their verbal and non-verbal reasoning scores and if at least one other sibling had a record of reading disability. Proband exclusion criteria included other disorders such as specific language impairment (SLI [MIM 606711]), autism (MIM 209850) or attention deficit-hyperactivity disorder (ADHD [MIM 143465]). These criteria identified some probands with high IQ scores and BAS scores within the ‘normal’ range. Therefore, after collecting 173 UK families the criteria were changed such that the proband's difference in their BAS single-word reading score had to be ≥1 SD below the population mean for their age-band (and not IQ), along with an IQ≥90, and the requirement of reading disability in another sibling dropped. A further 116 UK families were then collected with these new criteria. The total sample now comprises 289 UK families, with 685 siblings measured for a series of reading-related quantitative traits.

The 155 US families were drawn from the Colorado Learning Disabilities Research Center (CLDRC) twin study of reading disability. Twin pairs were identified from the records of 27 Colorado school districts and ascertained if at least one member had a school history of reading difficulty. Monozygotic twins were excluded, but additional non-twin siblings were included. Each child was assessed for a series of psychometric measures as detailed previously [23].

Briefly, the psychometric measures include graded tests for single-word reading (READ) and spelling (SPELL), and tests for orthographic coding by an irregular word task (OC-irreg; only UK families) and forced choice task (OC-choice; a pseudohomophone detection task), phoneme awareness (PA), phonological decoding (PD).

We also report a new collection of 317 UK cases of DD recruited through the Dyslexia Research Centre clinics in Oxford and Reading, and the Aston Dyslexia and Development clinic in Birmingham. These cases are between 8 and 18 years old, have a BAS2 single-word reading score ≤100 (at chronological age) and >1.5 SDs below that predicted by IQ.

Population controls were taken from the Human Random Control (HRC) panel of the European Collection of Cell Cultures (ECACC). We analysed the DNA of 287 unique samples from these cell lines. Three assumptions are made about these controls. Firstly, that ∼5% have DD due to the prevalence of this disorder in the UK. Secondly, that they are unrelated to our DD individuals, which is reasonable as they have been randomly ascertained from the UK and Ireland. Thirdly, that they come from the same ethnic origin as our DD samples, which is important to prevent population stratification from affecting our association analyses.

### Genotyping

Highly polymorphic microsatellite markers were genotyped by semi-automated fluorescent genotyping techniques with the ABI3700 machine and Genotyper® software from Applied Biosystems as previously described [23].

SNPs were genotyped with GoldenGate assays from Illumina® [36] or iPLEX assays from SEQUENOM® [37], [38], according to the manufacturers' instructions. Two GoldenGate assays of 1,536 SNPs were created for genotyping in a single multi-plex reaction. After amplification, hybridisation and washing steps, the arrays were scanned and analysed to generate genotypes which were then verified by eye. SEQUENOM®'s Assay Design software was used to design the PCR and extension primers for each SNP after downloading and processing sequences with Biomart and RealSNP, respectively. Genotypes were called automatically and verified by eye using SEQUENOM®'s Typer software.

### Gene and SNP Selection

The SNP selection and genotyping were conducted in two stages. Both stages utilised the International HapMap Project (IHMP) genotype data from 30 Centre d'Etude du Polymorphisme Humain (CEPH) trios to guide SNP selection (see Table S1). Our second stage superseded the first as more genotype data were then available from the IHMP, thereby increasing the number of available polymorphisms to test and the resolution of linkage disequilibrium. Exclusion criteria for the IHMP SNPs were deviations from Hardy-Weinberg (H–W) equilibrium (p-value<0.001), low genotype call rates (≤50%), Mendelian inheritance errors (>0) and low minimum allele frequency (<5%). HAPLOVIEW [39] created blocks of SNPs in strong LD according to the definition of Gabriel et al. [40], and subsequently selected haplotype-tagging SNPs (htSNPs) within each block to tag all haplotypes ≥3% frequency. htSNPs from all “genic blocks” in the candidate region were selected for genotyping, where we define a “genic block” as a Gabriel block covering any part of a gene (including any additional upstream or downstream sequence). Three genes remained totally uncovered by any block, whilst all others were covered completely or partially by at least one block. Details for all genes in the candidate region were downloaded from the University of California, Santa Cruz (UCSC) Genome Table Browser freezes July 2003 and May 2004, for the first and second stages, respectively (see Table S2).

### Data handling and analysis

The Integrated Genotyping System (IGS) [41] was used to store and check genotypes for Mendelian inheritance problems. MERLIN (1.1.1) [42] was used to detect unlikely double recombinants which might indicate further genotyping errors. PEDSTATS (0.6.9) [43] was used to assess levels of H–W equilibrium. STRUCTURE [44], [45] was used to assess population structure within and between sample sets by comparing the genotypes of 28 SNPs at genomic loci unlinked to DD. STRUCTURE was executed with a burn-in length of 1,000,000 followed by 1,000,000 iterations until completion. Analyses were performed within and between each sample set assuming K = 1 sub-populations (i.e. no population stratification), and K = 2 and 3 sub-populations (assuming a model of admixture) and revealed a homogenous ancestry of samples.

### Linkage analysis

Multi-point linkage analysis was performed. GENEHUNTER (2.1_r2 beta) [46] was used to apply the traditional Haseman-Elston (HE) sibling-pair squared trait-differences model [47] or a variance components (VC) framework without dominance variance and with a single-trait mean. The DeFries-Fulker (DF) regression technique [48] was applied with scripts and macros for the SAS package [49].

### Association analyses

Family-based samples with their quantitative traits were analysed with the 'total association' option within QTDT (2.5.1) [50], [51]. The association tests were not adjusted for linkage. All traits were tested against each SNP individually. Qualitative population-based analyses were performed with PLINK (1.01) [52] which supports the genotype and allele count tests. Quantile-quantile plots validated these tests (see Figure S1).

## Results

### Linkage analysis of DD susceptibility on chromosome 18

The 89 UK families and 116 (of the 119) US families used for the original whole genome-wide scans and the 84 UK families used for replication [23], were genotyped with a denser set of microsatellite markers. We detected the same linkages as previously reported (see Figures S2 and S3). Subsequently, we genotyped a third set of 91 UK families and a second set of 39 US families with microsatellite markers to the same high density.

Both new samples reveal linkage at 18q12.2; the 91 UK families with OC-irreg (LOD≈1.5; see Figure S2) and the 39 US families with OC-choice (LOD≈3.5; see Figure S3). Linkage at 18p11.2 was also observed in the 39 US sample with PD, SPELL and OC-choice (LOD≈1.5) and also at 18q21 with PD and READ (LOD>3.5). Combining all 264 UK families together produced linkage at 18q12 with a LOD>2 and at 18p11.2, 18q12.2 and 18q22.3 with a LOD≈1.5 with various traits (see Figure S2).

**Figure 1:**
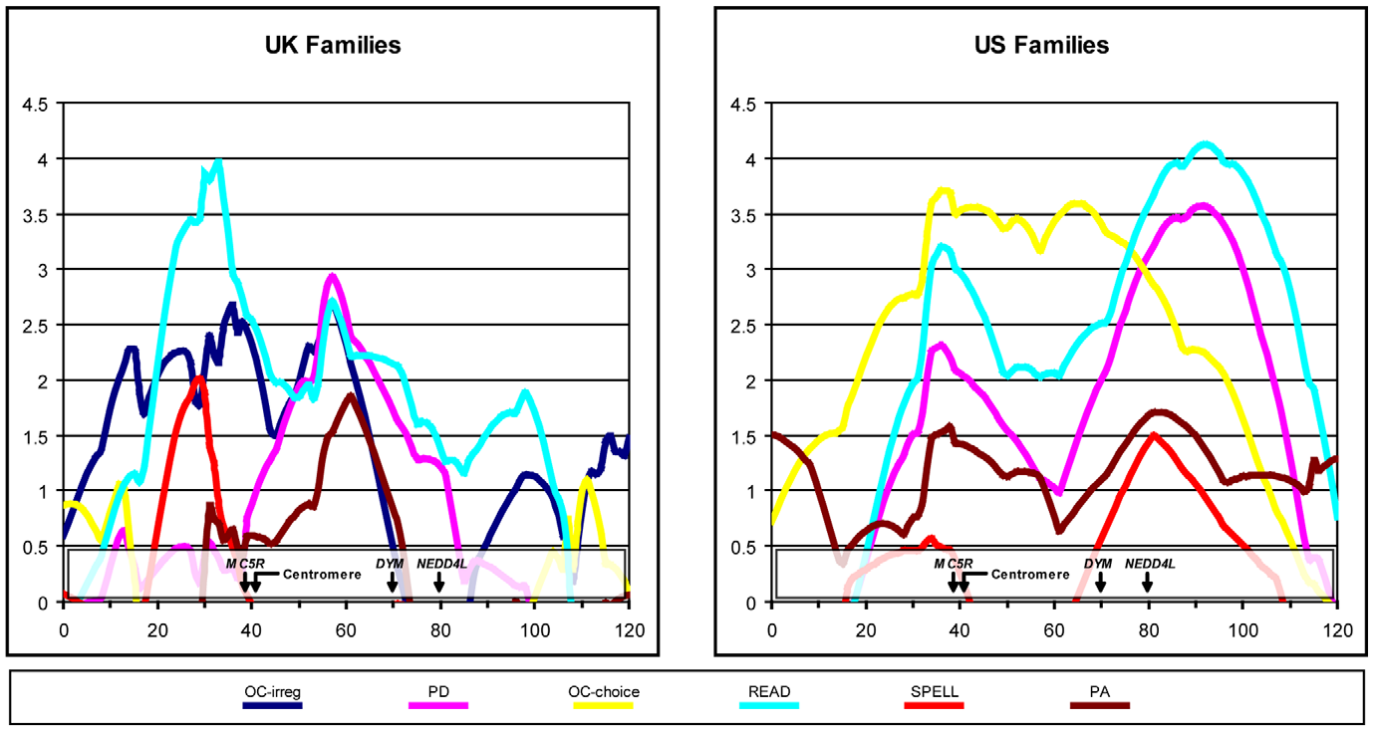
DF linkage analysis on chromosome 18 of the UK and US families. Families were selected for each quantitative trait if any sibling scored >1.5 SD or >2 SD below the normative mean for that trait, for the UK or US families, respectively. The units of the x-axis are cM and y-axis are negative t-scores.

For analysis with the DF regression technique, UK or US families were selected if at least one child scored >1.5 SDs or >2.0 SDs, respectively, below the normative mean for any one of the reading-related traits (see Figure 1). This selection yielded 188 UK families and 133 US families. DF analysis revealed strong linkage at 18p11.2 and 18q12.2 with READ in both the UK and US families, and also at 18q22.1 in the US families.

This linkage analysis therefore provides supporting evidence of a DD susceptibility gene on chromosome 18. However, we were unable to narrow down the region and instead extended the linkage to the other side of the centromere. We therefore carried out a high-throughput genotyping and association strategy of all genic regions in the broader candidate region spanning 18p11.31 to 18q21.31. A total of 768 samples, including quality control samples, were genotyped in 8×96 well plates, which represented the most cost-effective strategy. To enable family-based tests, the 188 UK families that gave strongest linkage to chromosome 18 with the DF analysis were selected for genotyping. Following QC procedures one family was removed. The remaining 187 UK families included 68 from the original 89 families used in the whole genome-wide linkage scan, 53 from the 84 families used in the replication of *DYX6*, and 66 from 91 families newly reported here.

### Association analysis: discovery stage

Altogether, 2,895 SNPs in our candidate region were successfully genotyped in 759 samples, and of these >97% SNPs and >99% samples had a genotype call rate >96%. The 759 samples included 187 successfully genotyped UK families known hence forth as the “discovery sample” (see Table 1).

**Table 1:** Summary of the independent samples and tests performed for the association study on chromosome 18.

		Analysis type	# tests
Discovery sample	187 UK families	QTDT	6×2895
Replication sample 1	102 UK families	QTDT	6×24
Replication sample 2	152 US families	QTDT	5×24
Replication sample 3	317 UK DD cases and 287 controls	Allelic & genotypic tests	2×24

A summary of the samples used and the tests conducted in the discovery and replication stages of the association study on chromosome 18. Note, an additional 25 UK families not genotyped for microsatellites, were used in the replication stage of SNP associations, and 3 US families genotyped for microsatellites in the linkage stage failed genotyping for the association stage.

Quantitative association analyses with each of the six quantitative traits were performed on all 2,895 SNPs in the discovery sample (see Figure S4 and Table S3 for the complete results, and Table S4 for the top results). The most significant associations (p-values = 0.0003) were with rs7507114 and rs4800148 in the genes “chromosome 18 open reading frames 1” (*C18orf1* [MIM 606571]) and “Cdk5 and Abl enzyme substrate 1” (*CABLES1* [MIM 609194]), respectively. The top associations consistently showed the same direction for each quantitative trait tested, in agreement with our observations of multivariate linkage analysis [24].

### Association analysis: Replication stage

The 11 most significant SNPs (p-values<0.001) from the discovery stage were selected for replication in independent samples. Another 14 highly ranked SNPs (p-values between 0.001 and 0.002) were also selected if they were compatible with the iPLEX assays. In total, 25 SNPs were selected for further genotyping (see Table 2) in our independent samples consisting of 102 UK families, 152 US families and 317 UK DD cases and 287 UK population controls (see Table 1). Tests in the family samples were performed with QTDT whilst tests in the cases and controls were performed with PLINK (see Tables S5 & S6 for the complete results).

**Table 2:** SNPs selected for further genotyping in independent samples.

SNPs selected for replication^a^	Block #	Gene	Ranking^b^
rs7507114	1015	C18orf1, C18orf15	1
rs4800148	1121	CABLES1	1
rs11659463	2162	SMAD2, MADH2	3
rs17802670	256	DLGAP1	4
rs17439829	559	LAMA1	5
rs9957285	2094	LOXHD1	5
rs11874896	449	EPB41L3	7
rs10502812	1936	AK131011	7
rs4464148	2246	SMAD7	7
rs3018202	1913	RIT2, U78166	10
rs7241007	N/A	ZNF519	10
rs6505873	1049	ZNF519	12
rs12607710	975	PTPN2	15
rs1299348	1033	MC5R	15
rs9958315^c^	1571	C18orf34	15^c^
rs1790480	2294	ACAA2, SCARNA17	15
rs506696	2296	MYO5B	15
rs11873029	N/A	FLJ20071, DYM	15
rs11661879	2240	KIAA0427	22
rs8083578	2654	NEDD4L	22
rs8094063	2346	MRO, B29	25
rs1941001	441	EPB41L3	28
rs8094327	2663	NEDD4L	30
rs555879	N/A	MYO5B	30
rs12606138	2664	NEDD4L	34

Displayed are the SNPs selected for further genotyping in the independent replication samples. The p-values for these SNPs can be found in Tables S3 & S4.

a Some SNPs were replaced by proxy SNPs if the original were not compatible with the assays designed for the iPLEX genotyping system;

b ranking only given for those SNPs selected for genotyping in replication samples. Equally ranked SNPs are given the same rank number, and subsequent rank numbers are skipped to compensate;

c This SNP (rs9958315) failed genotyping in the replication samples.

Significant results were observed in the same direction as the discovery sample for 5 SNPs with the QTDT analysis and 5 SNPs with either of the population-based analyses (p-values<0.05; see Table 3). Of particular note are two SNPs that gave significant results in both the 102 UK families and the case-control samples; rs1299348 within *MC5R* and rs11873029 within *DYM*. Also of note are the two SNPs rs8094327 and rs12606138 within *NEDD4L* that both replicated in the case-control samples.

**Table 3:** Results for any SNPs that replicated in any independent sample.

		Association analysis for minor allele^a^			
SNP	Gene	Discovery families^b^	102 UK families^b^	152 US families^b^	317 UK DD cases and 287 population controls^c^
rs11874896	EPB41L3	[-] P<0.001 (PA)	[-] 0.05 (OC-irreg)	n/s	n/s
rs1299348	MC5R	0.006 (OC-irreg)0.005 (OC-choice)0.001 (READ)	0.04 (SPELL)	n/s	0.009 (allelic)0.009 (genotypic)
rs10502812	AK131011	[-] P<0.001 (PD)	n/s	−0.05 (PD)−0.03 (SPELL)	n/s
rs11661879	KIAA0427	0.03 (OC-irreg)0.001 (SPELL)0.02 (PA)	0.04 (SPELL)	n/s	n/s
rs11873029	DYM	0.004 (PD)0.001 (PA)	0.01 (OC-choice)	n/s	0.006 (allelic)0.02 (genotypic)
rs555879	MYO5B	0.03 (PD)0.02 (SPELL)0.002 (PA)	n/s	n/s	0.03 (genotypic)
rs8094327	NEDD4L	0.02 (OC-irreg)0.008 (PD)0.002 (READ)0.04 (SPELL)	n/s	n/s	0.007 (allelic)0.007 (genotypic)
rs12606138	NEDD4L	0.02 (OC-irreg)0.007 (PD)0.002 (READ)0.04 (SPELL)	n/s	n/s	0.005 (allelic)0.002 (genotypic)

a absolute values presented are p-values for the minor allele, and the negative sign [-] indicates an association of risk for DD with that allele, else that allele confers protection against DD. n/s means not significant (p-value>0.05) – the actual p-values are available in the supplementary tables;

b the acronyms OC-irreg, PD, OC-choice, READ, SPELL and PA in brackets refer to the trait giving association with QTDT;

c the terms allelic or genotypic in brackets indicate the allelic or genotypic test for that p-value.

## Discussion

In the present study we confirm linkage to DD on chromosome 18 by genotyping an extended sample of our UK and US families with a denser set of microsatellite markers. By combining all UK or US families, the linkage signals appear broad (>40 Mb) and span the centromere from 18p11.2 to 18q12.2.

**Figure 2:**
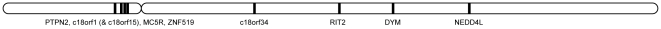
Location of interesting genes on chromosome 18. The location of the genes that showed some evidence of replication are highlighted along chromosome 18.

We then performed a high-throughput SNP genotyping experiment covering genes from 18p11.31 to 18q21.31 in a subset of 187 UK families. Highly associated SNPs were then genotyped in the remaining independent samples; 102 UK families, 152 US families, 317 UK DD cases and 287 UK controls (see Table 1). We successfully found associations for SNPs within several genes, particularly *MC5R*, *DYM* and *NEDD4L*, with the same trend in independent samples. Between samples, the associations were not always with the same trait or test, and so not all are replications in the purest sense. However, we know from the multivariate linkage analysis that all six traits are influenced by the same underlying QTL(s) on chromosome 18. Furthermore, there is consistency between the observed linkage and association patterns. The linkage signals in both UK and US samples are spread across chromosome 18, spanning some 40 Mb, and accordingly we find associations to genes located along this chromosome (see Figure 2). Hence, it would be attractive to speculate that this spread of associations across the candidate region explains the broad spread of linkages. Indeed, READ gives strongest linkage to 18p11.21 in the discovery sample (see Figure 1), and the genes *PTPN2*, *C18orf1*, *C18orf15*, *MC5R* and *ZNF519* at 18p11.21 each appear strongly associated with READ (see Table S4). Whilst on the q-arm, strongest linkage is at 18q12.2 in the discovery sample with PD, READ, OC-irreg, and PA, and the genes *C18orf34* and *RIT2*, at 18q12.1 and 18q12.3 respectively, are associated strongest for all or most of these traits. Also on the q-arm, at 18q21, are *DYM* and *NEDD4L*, and together these are strongly associated to these traits too.

*MC5R* is a G-protein-coupled 7 transmembrane receptor [53] that binds melanocortins, including the neuropeptides adrenocorticotropic hormone (ACTH) and α-, β- and γ-melanocyte-stimulating hormones (α-,β- and γ-MSH). The melanocortins are involved in skin physiology, behaviour, learning and memory [54], [55], [56]. ACHT, or peptides derived from it, are implicated in attention, visual attention, analytical thinking, spatial awareness and musical ability [57], [58], [59], [60]. *MC5R* is a single exon gene less than 1 kb in length. Here, we find association to the SNP rs1299348 within this gene in 3 independent samples. The major allele of ∼65% frequency confers risk to DD susceptibility.

*DYM* is nearly 500 kb long, and mutations in this gene cause Dyggve-Melchior-Clausen syndrome [MIM 223800] [61], characterised by short trunk dwarfism, developmental delay, microcephaly and psychomotor retardation. We find here an association to a single SNP, rs11873029, in the discovery sample. We then found association to a proxy SNP in our independent UK families and UK case and control samples. The two SNPs are found in separate introns of *DYM*, and the major alleles of ∼85% frequency confer risk to DD susceptibility.

*NEDD4L* has 78% amino acid sequence identity with neural precursor cell expressed, developmentally down-regulated 4 (*NEDD4* [MIM 602278]) and is implicated in the regulation of the epithelial sodium channel [62]. A SNP within *NEDD4L* (rs4149601) has been separately associated to ADHD [63], hypertension and blood pressure [64], [65], [66], [67], and another SNP (rs2288774) has also been associated with ADHD [63]. DD shows strong co-morbidity with ADHD [68] and has also been tentatively associated to low blood pressure [69]. Proxies of these two SNPs that were genotyped in the discovery sample did not reveal association with DD. However, a marked haplotypic diversity has previously been observed within *NEDD4L* such that opposite alleles of the same SNPs associate with hypertension in different white populations [65], [70]. *NEDD4L* is about 350 kb in length, and we identify here three associated intronic SNPs in our discovery sample that are separated by about 135 kb. Two of these SNPs replicated in our case and control samples (see Table 3). The major alleles, of 70–80% frequency, for each of these SNPs appear to confer risk for DD susceptibility.

The power of this study was limited by a relatively small sample size. Another limitation was the failure of some SNPs to genotype and the technical constraints of genotyping other SNPs, as this has led to some genetic variability remaining untested. A further issue not yet addressed is that of multiple-testing. The p-values reported here are all uncorrected for multiple-testing. Given that 2,895 SNPs and six quantitative traits were analysed in the discovery stage analysis, a total of 17,370 tests were performed in the discovery sample (see Table 1). In the follow-up replication stages, 144 tests were performed in the independent UK families, 120 tests in the US families and 48 tests with the independent UK cases and controls. A Bonferroni corrected significance threshold for the discovery stage is therefore 2.87×10^−6^, and for the replication stages are 3.47×10^−4^ (UK families), 4.17×10^−4^ (US families) and 1.04×10^−3^ (UK cases and controls). None of the SNPs reached this level of significance in either the discovery or replication stages. However, a simple Bonferroni correction is highly conservative here as many of the SNPs are in strong LD with each and the traits themselves are highly correlated.

We also recognise that our association results were not as consistent or significant as the linkage study, and this may in part be due to rare variations present in individual families. However, with our approach we did find consistent association to genetic variants in several independent samples for *MC5R*, *DYM* and *NEDD4L*. Published biological evidence for these genes make them attractive candidates with respect to DD susceptibility. We suggest that further independent samples be tested for these genes.

### Web Resources

Biomart, http://www.biomart.org

HapMap homepage, http://www.hapmap.org/

Online Mendelian Inheritance in Man (OMIM), http://www.ncbi.nlm.nih.gov/Omim/

RealSNP, Sequenom, https://www.realsnp.com/

UCSC Genome browser: http://www.genome.ucsc.edu/

## Supporting Information

Figure S1 Quantile-Quantile plots of the association analyses. Quantile-quantile plots were created for the association analyses performed in QTDT and TDT. The left column displays the SNPs from the first stage, the middle displays the SNPs from the second stage, and the right displays both stages combined. The x-axis is the expected test statistic and the y-axis is the observed test statistic.
(0.47 MB TIF)

Figure S2 HE and VC linkage analysis on chromosome 18 in the UK families. The HE and VC linkage analyses were performed in the three sets of independent UK families separately and combined. The units of the x-axis are cM and y-axis are LOD scores.
(0.39 MB TIF)

Figure S3 DF linkage analysis on chromosome 18 of the US families. The DF linkage analyses was performed in the two sets of independent US families separately and combined. Families were selected for each trait if any sibling scored >2 SD below the normative mean for that trait. The units of the x-axis are cM and y-axis are negative t-scores.
(0.16 MB TIF)

Figure S4 Association analysis with the discovery sample of 187 UK families. Association analyses were performed by QTDT with the six quantitative traits. Results are shown with respect to the minor allele of each SNP.
(0.15 MB TIF)

Table S1 Information on the SNPs in the candidate region downloaded from the IHMP. Contains details on the SNPs genotyped in the CEPH trios and downloaded from the IHMP in phases I and II for the chromosome 18 DD susceptibility candidate region (18p11.2 to 18q12.2), for stages 1 and 2 respectively.
(0.02 MB XLS)

Table S2 Selection of genes on chromosome 18 in the candidate region. A complete list of all the genes in the candidate region, including their physical co-ordinates, Gabriel block coverage, and the number of htSNPs identified for genotyping.
(0.04 MB XLS)

Table S3 Complete results of the association analyses. Listed are the complete results of the QTDT analysis of the 2,895 SNPs genotyped in the discovery sample of 187 UK families.
(0.69 MB XLS)

Table S4 Results from the QTDT association analysis with the discovery sample of 187 UK families. Displayed are the SNPs selected for genotyping in independent samples, including all SNPs achieving a nominal p-value<0.001 for any quantitative trait and other high ranking SNPs. Also displayed are any p-values<0.05.
(0.02 MB XLS)

Table S5 Complete results of the association analysis with QTDT in the independent UK and US families. Listed are the complete results of the association analysis performed in QTDT with the independent UK and US families. Also shown are the results of the analysis performed in all the UK families combined.
(0.03 MB XLS)

Table S6 Complete results of the association analysis in the independent UK DD cases and population controls. Listed are the complete results of the association analysis performed with the independent UK cases and population controls.
(0.02 MB XLS)
