# Correction: Identification of Candidate Genes for Dyslexia Susceptibility on Chromosome 18

**DOI:** 10.1371/annotation/2294a38b-878d-42f0-9faf-0822db4a0248

**Published:** 2010-12-15

**Authors:** Thomas S. Scerri, Silvia Paracchini, Andrew Morris, I. Laurence MacPhie, Joel Talcott, John Stein, Shelley D. Smith, Bruce F. Pennington, Richard K. Olson, John C. DeFries, Anthony P. Monaco, Alex J. Richardson

The authors wish to add an author to the byline. The 12th author is Alex J. Richardson. His affiliation is The Department of Social Policy and Social Work, University of Oxford, Oxford, United Kingdom.

