# Supplementary figures and images for: Identification of Candidate Genes for Dyslexia Susceptibility on Chromosome 18
Source: PLoS One. 2010 Oct 28;5(10):e13712. doi: 10.1371/journal.pone.0013712 (PMC2965662; doi:10.1371/journal.pone.0013712)

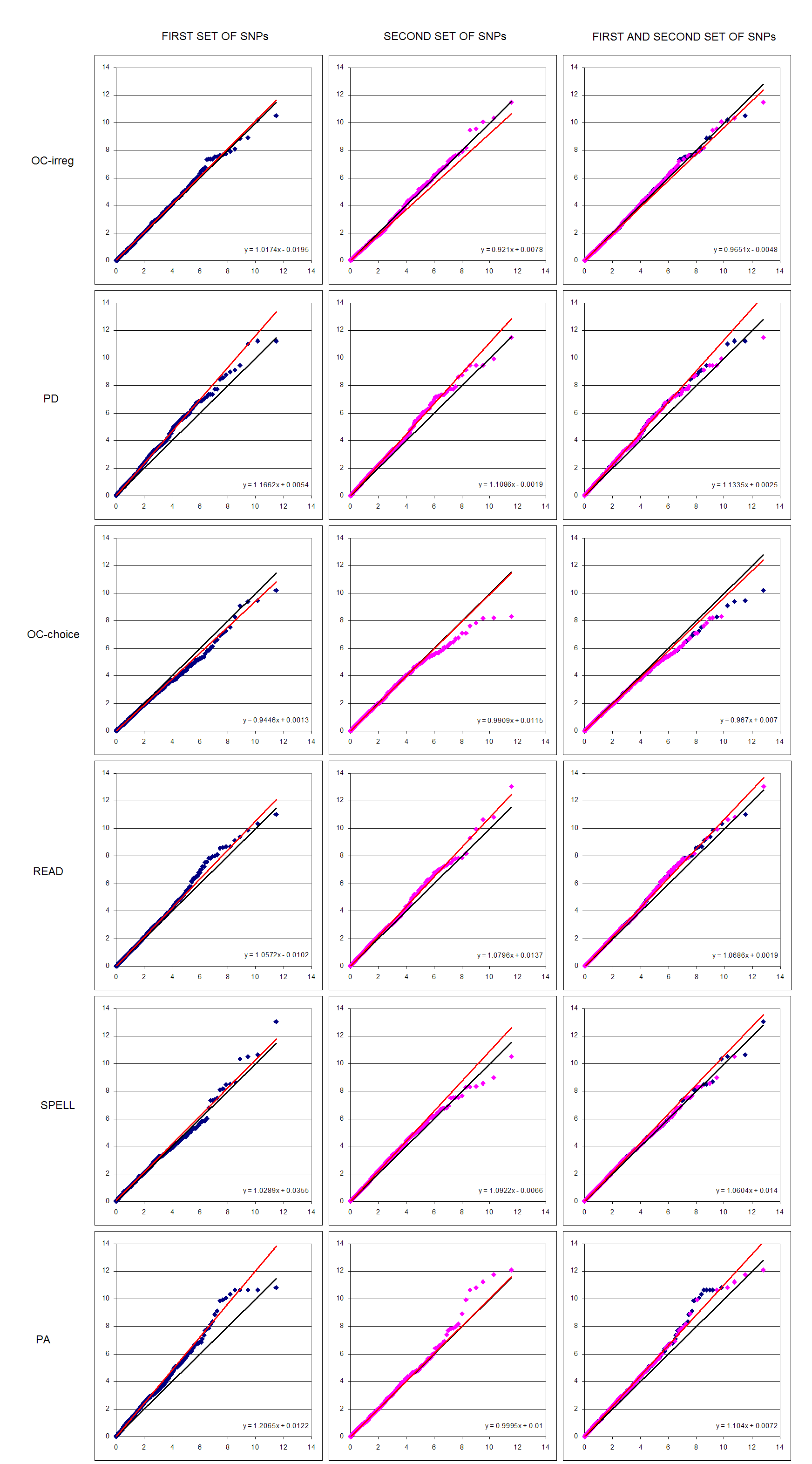

Supplement: Figure S1 — Quantile-Quantile plots of the association analyses. Quantile-quantile plots were created for the association analyses performed in QTDT and TDT. The left column displays the SNPs from the first stage, the middle displays the SNPs from the second stage, and the right displays both stages combined. The x-axis is the expected test statistic and the y-axis is the observed test statistic. (0.47 MB TIF) [file pone.0013712.s001.tif]

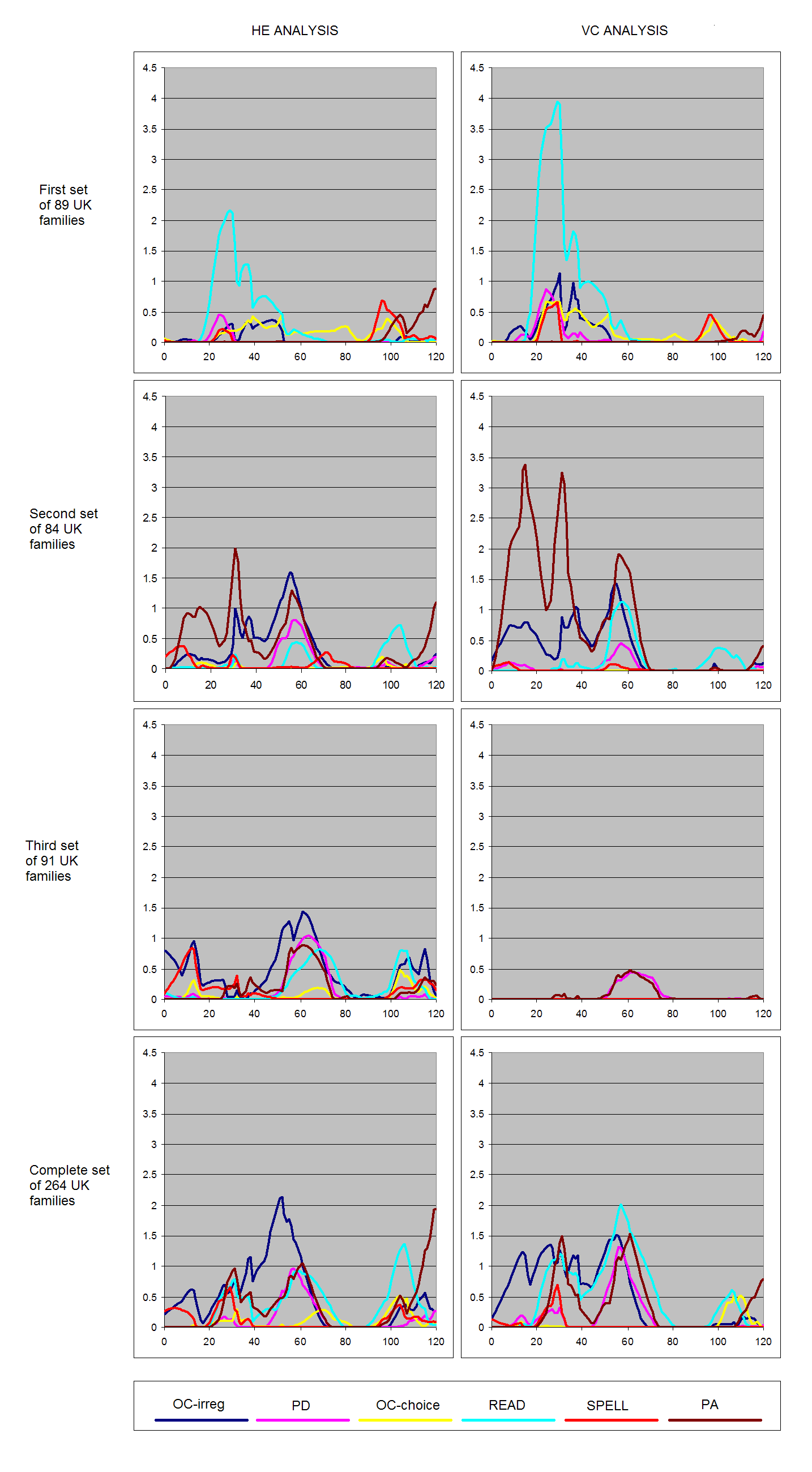

Supplement: Figure S2 — HE and VC linkage analysis on chromosome 18 in the UK families. The HE and VC linkage analyses were performed in the three sets of independent UK families separately and combined. The units of the x-axis are cM and y-axis are LOD scores. (0.39 MB TIF) [file pone.0013712.s002.tif]

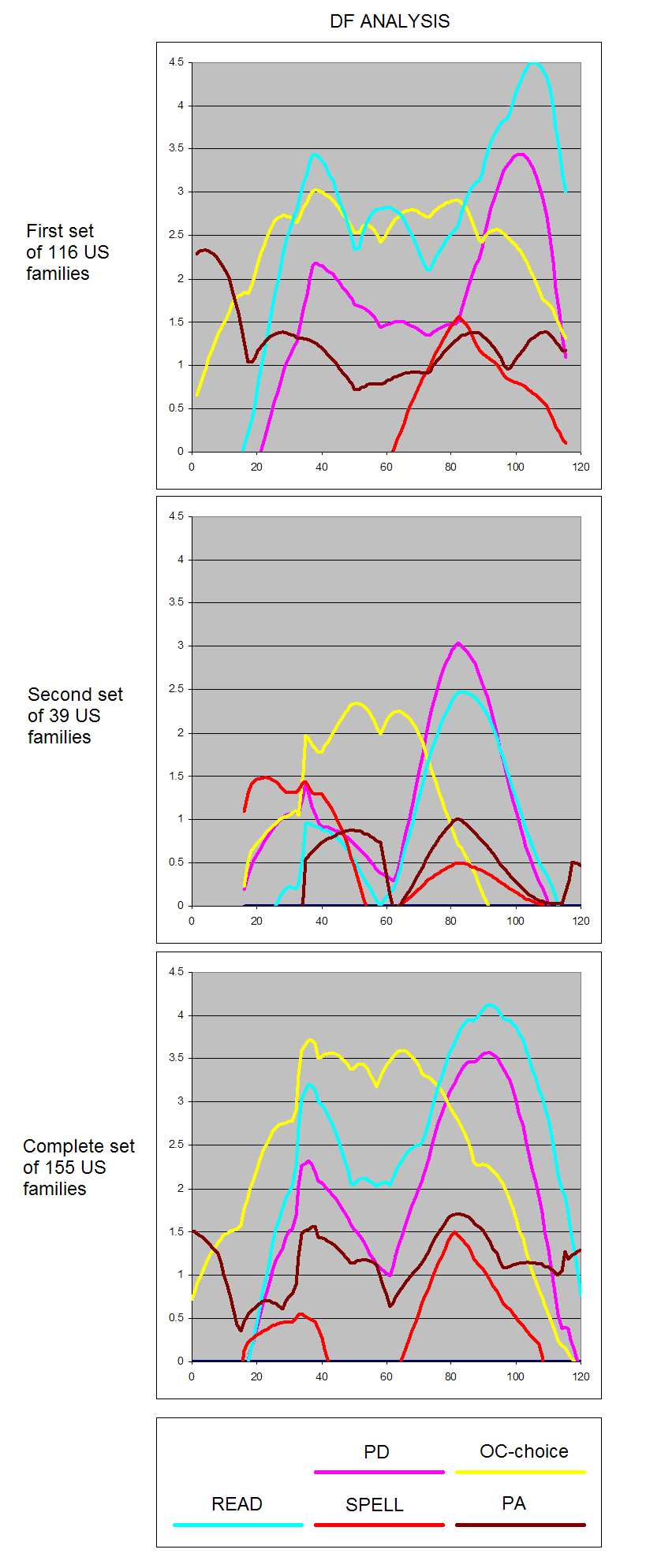

Supplement: Figure S3 — DF linkage analysis on chromosome 18 of the US families. The DF linkage analyses was performed in the two sets of independent US families separately and combined. Families were selected for each trait if any sibling scored >2 SD below the normative mean for that trait. The units of the x-axis are cM and y-axis are negative t-scores. (0.16 MB TIF) [file pone.0013712.s003.tif]

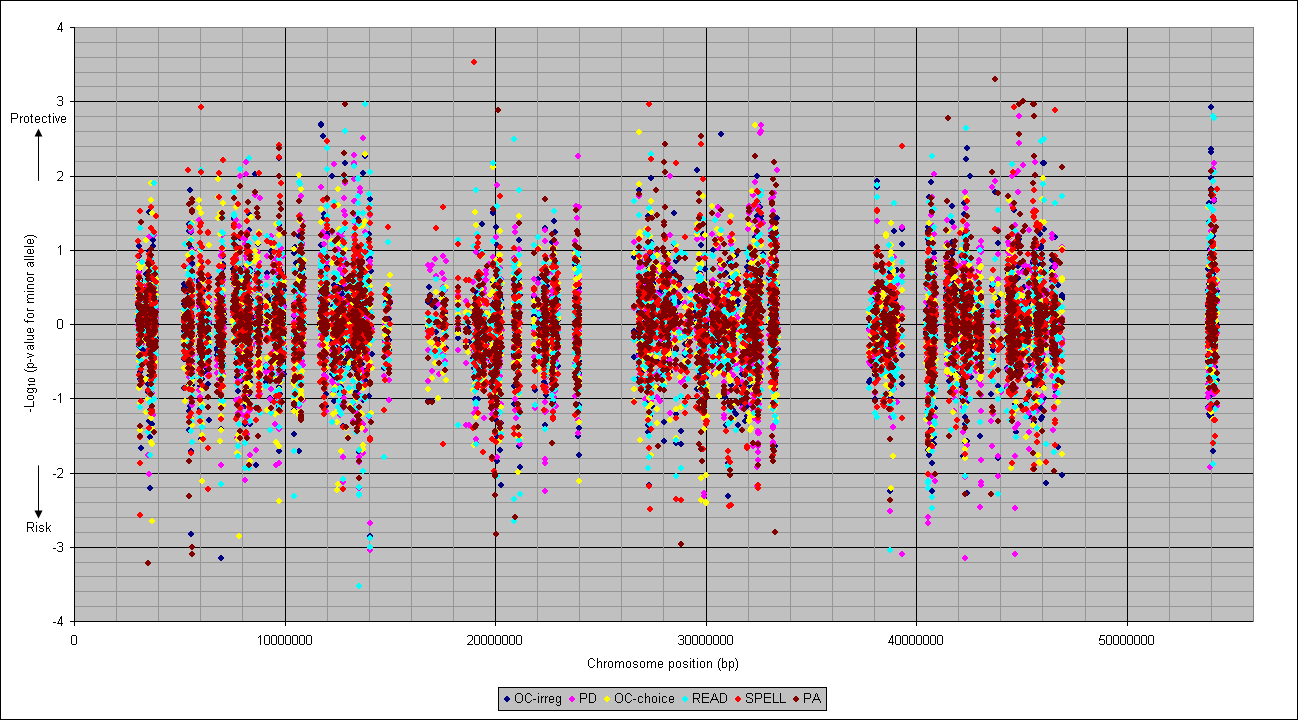

Supplement: Figure S4 — Association analysis with the discovery sample of 187 UK families. Association analyses were performed by QTDT with the six quantitative traits. Results are shown with respect to the minor allele of each SNP. (0.15 MB TIF) [file pone.0013712.s004.tif]
